# Prenatal Management of Spinal Muscular Atrophy in the Era of Genetic Screening and Emerging Opportunities in In Utero Therapy

**DOI:** 10.3390/biomedicines13081796

**Published:** 2025-07-22

**Authors:** Silvestar Mežnarić, Andrej Belančić, Valentino Rački, Dinko Vitezić, Jasenka Mršić-Pelčić, Kristina Pilipović

**Affiliations:** 1 Department of Basic and Clinical Pharmacology and Toxicology, Faculty of Medicine, University of Rijeka, 51000 Rijeka, Croatia; silvestar.meznaric@medri.uniri.hr (S.M.); andrej.belancic@medri.uniri.hr (A.B.); dinko.vitezic@medri.uniri.hr (D.V.); jasenka.mrsic.pelcic@medri.uniri.hr (J.M.-P.); 2 Department of Neurology, Faculty of Medicine, University of Rijeka, 51000 Rijeka, Croatia; valentino.racki@medri.uniri.hr; 3 Department of Neurology, Clinical Hospital Centre Rijeka, 51000 Rijeka, Croatia

**Keywords:** spinal muscular atrophy, fetal therapies, prenatal diagnosis, genetic testing

## Abstract

Spinal muscular atrophy (SMA) is a severe autosomal recessive neuromuscular disorder and a leading genetic cause of infant mortality. Advances in disease-modifying therapies have significantly improved outcomes when treatment is initiated early, underscoring the importance of timely diagnosis. With the growing availability of prenatal genetic screening and high-resolution molecular diagnostics, opportunities for early detection, and potentially in utero intervention, are rapidly expanding. This narrative review synthesizes current evidence on the prenatal management of SMA, focusing on diagnostic strategies, the clinical application of fetal genetic testing, and the emerging potential of fetal therapy. We explore both invasive and non-invasive diagnostic approaches and evaluate experimental prenatal treatment modalities, while critically addressing the associated ethical, regulatory, and economic considerations. As the field progresses, integrating in utero strategies into clinical care may reshape perinatal medicine and offer transformative potential for genetic neurodegenerative disorders diagnosed before birth. The convergence of early diagnosis, fetal intervention, and personalized genetic counseling will be central to optimizing care pathways and outcomes in the era of precision medicine. Although significant challenges remain, the translation of fetal therapy into routine clinical practice is approaching feasibility. Future clinical trials, anchored in definitive prenatal diagnosis, will be essential, with benefits potentially outweighing the inherent procedural risks.

## 1. Introduction

Spinal muscular atrophy (SMA) is a rare autosomal recessive neuromuscular disorder caused by progressive degeneration of alpha motor neurons in the anterior horn of the spinal cord, leading to symmetric muscle weakness and atrophy. With an estimated incidence of approximately 1 in 10,000 live births and a prevalence of 1–2 per 100,000 individuals, SMA is among the most clinically significant inherited neurological disorders identified in infancy [1,2].

The condition is most often attributable to a homozygous deletion or mutation in the *SMN1* gene, which encodes the survival motor neuron (SMN) protein—an essential factor for motor neuron maintenance and function. The *SMN1* and *SMN2* genes lie within the telomeric and centromeric halves, respectively, of a large, inverted duplication on chromosome 5q13 [3,4]. Insufficient levels of SMN protein result in selective motor neuron vulnerability and gradual neuromuscular decline. The disease phenotype is partially modulated by the copy number of *SMN2*, a closely related gene that produces only small amounts of functional SMN protein due to alternative splicing. While unable to fully compensate for the loss of *SMN1*, the *SMN2* copy number serves as a useful prognostic biomarker and is increasingly used to guide treatment decisions [5,6].

SMA presents as a clinically heterogeneous disorder, with symptom severity and progression varying widely among individuals. This variability is largely determined by the age at onset, the maximum motor milestone achieved, and the rate of neuromuscular decline. Traditionally, in the era preceding disease-modifying therapies (DMDs), SMA was classified into five clinical types (0–4), reflecting a continuum from the most severe prenatal presentations to milder adult-onset forms (Table 1). The phenotypic spectrum ranges from infants with type 0 SMA, who show signs in utero and typically do not survive beyond the neonatal period, to individuals with type 4 SMA, who may present in early adulthood with mild proximal muscle weakness and retain functional ambulation throughout life [7,8,9].

In line with findings by Watihayati et al. [10], among patients with type I SMA, 71% carried two *SMN2* gene copies and 29% carried one, whereas in milder phenotypes (types II and III), 71% had three or more copies—specifically, 29% with two, 57% with three, and 14% with four copies—highlighting the inverse correlation between *SMN2* copy number and disease severity [10]. The therapeutic landscape of SMA has been fundamentally reshaped by the emergence of DMDs, which have substantially enhanced survival and motor function outcomes (Table 2) [11]. Nusinersen, an antisense oligonucleotide administered via intrathecal injection, was the first treatment to gain approval from the European Medicines Agency (EMA) in May 2017, supported by evidence from the ENDEAR and CHERISH trials [12,13,14]. Subsequently, onasemnogene abeparvovec, a systemic gene replacement therapy delivered intravenously, received conditional EMA approval in May 2020, which was elevated to full authorization in May 2022 following compelling data from the START and STR1VE studies [15,16,17,18]. Most recently, risdiplam, an orally bioavailable small molecule that modulates *SMN2* pre-mRNA splicing to enhance functional protein levels, was granted EMA approval in March 2021 after demonstrating clinical efficacy in the FIREFISH and SUNFISH trials [19,20,21].

The timing of DMD initiation is critical in SMA, as earlier treatment consistently yields superior motor and survival outcomes. This urgency stems from the rapid and progressive degeneration of alpha motor neurons in the spinal cord, which occurs in the early stages of the disease. Therefore, integration of newborn screening for 5q SMA into national health programs enabled presymptomatic diagnosis and timely commencement of therapy. Such early intervention then prevents irreversible motor neuron loss, reduces the risk of severe disability, respiratory complications, and mortality [22]. Building upon this framework, the prospect of prenatal screening and in utero therapy emerges as the next frontier. Recent advancements in maternal–fetal medicine, alongside the advent of highly sensitive and accessible genetic sequencing technologies, have made it possible to identify SMA and other genetic disorders during pregnancy. This earlier detection facilitates more informed genetic counseling and offers a crucial therapeutic window to intervene before the onset of irreversible neurodegeneration. Targeted prenatal treatments hold promise to not only prevent or attenuate disease progression but also improve the long-term neuromuscular function and quality of life of affected children [23].

A recent landmark case report described the first documented use of prenatal risdiplam therapy in a fetus diagnosed with SMA type 1 via prenatal genetic testing [24]. Administered off-label during the final six weeks of gestation as part of an interventional study, risdiplam was associated with the absence of clinical SMA manifestations more than two years after birth, suggesting durable therapeutic benefit and a favorable safety profile [23,24]. This unprecedented outcome challenges the conventional postnatal treatment paradigm and supports the hypothesis that earlier, in utero intervention may maximize clinical outcomes and mitigate long-term disability. 

Prompted by this breakthrough, the present narrative review synthesizes the emerging, but still limited, evidence on prenatal screening and fetal therapy for SMA. A comprehensive literature search was conducted via PubMed/MEDLINE using the terms (spinal muscular atrophy OR SMA) AND (prenatal OR in utero). A narrative synthesis approach was adopted to integrate findings across heterogeneous study designs and publication types. This method allowed for a contextualized interpretation of emerging data, highlighting methodological variability and gaps in the literature.

This review aims to explore the therapeutic potential of agents such as risdiplam, examine the clinical and ethical implications of prenatal testing, discuss challenges associated with in utero treatment, and outline future directions for research and policy. It argues that the integration of early diagnosis, fetal intervention, and personalized genetic counselling will be central to transforming care pathways and outcomes in the era of DMDs.

## 2. Prenatal Testing for SMA—Benefits vs. Risks

Prenatal screening for SMA offers benefits and risks for high-risk pregnant women with a family history. SMA carrier screening can identify couples at risk of having affected offspring, enabling informed decision making and genetic counseling [25,26]. The American College of Obstetricians and Gynaecologists recommends offering SMA carrier screening to all pregnant women [26]. This section analyses contemporary molecular methods used in the prenatal diagnosis of SMA, providing an overview of key studies, detailing the advantages and limitations of invasive and non-invasive approaches, and suggesting guidelines for future research.

### 2.1. Invasive Methods

When referring to invasive methods, the invasive component involves the sampling of biological material that poses risks to both the mother and fetus. Sampling is performed through two primary approaches: chorionic villus sampling (CVS) or amniocentesis [27].

#### 2.1.1. Chorionic Villus Sampling

CVS represents a form of prenatal diagnosis conducted to determine chromosomal or genetic disorders in the developing fetus [28]. This particular sampling technique typically occurs between 10 and 12 weeks of gestation, which is earlier than amniocentesis or percutaneous umbilical cord blood sampling, making it the preferred technique before 15 weeks. CVS was first performed in Milan by Italian biologist Giuseppe Simoni, scientific director of the Biocell Center, in 1983 [29]. Use as early as eight weeks has been described in special circumstances, although this is not routinely recommended [30]. The procedure can be performed in either a transcervical or transabdominal manner, depending on placental location and other factors [31]. Although this procedure is most commonly associated with testing for Down syndrome (trisomy 21), CVS can detect virtually all aneuploidies including Edwards’ syndrome (trisomy 18), Patau syndrome (trisomy 13), Turner syndrome, Klinefelter syndrome, and other sex chromosome abnormalities, as well as structural aberrations such as deletions, duplications, translocations, and inversions [32]. Furthermore, it enables diagnosis of microdeletion syndromes such as DiGeorge, 1p36, Cri-du-Chat, and Prader–Willi/Angelman syndromes, among others. When there is a known mutation in the family or clear indication, CVS can detect numerous monogenic disorders, including cystic fibrosis, thalassemia, hemophilia, Duchenne muscular dystrophy, SMA, phenylketonuria, metabolic diseases, and many others [32,33]. Chorionic villus sampling is associated with several potential risks, the most significant being miscarriage, with recent studies estimating the procedure-related risk at approximately 0.5% to 1%, which is similar to that of amniocentesis [34]. Other complications include vaginal bleeding, which occurs in up to 10% of cases, and transient abdominal pain at the puncture site [30]. Infection is a rare but recognized risk following CVS, as with any invasive procedure [35]. Performing CVS before 10 weeks of gestation has been linked to an increased risk of fetal limb reduction defects, which is why the procedure is generally recommended after this gestational age [28].

Additional risks include amniotic fluid leakage or premature rupture of membranes, which are uncommon, and the possibility of obtaining an inadequate or uninformative sample in about 1% of cases, sometimes necessitating repeat testing or subsequent amniocentesis [31]. Another consideration is confined placental mosaicism, observed in approximately 1% of cases, which may not reflect the true fetal karyotype and can complicate the interpretation of results [36,37]. Several researchers have questioned the potential association between chorionic villus sampling and the subsequent development of preeclampsia and eclampsia; however, the results did not show an increased risk [38]. Rh sensitization is also a potential risk for Rh-negative mothers, but this can be prevented with appropriate prophylaxis. Overall, while CVS is a valuable sampling tool, these risks should be carefully discussed with patients before the procedure.

#### 2.1.2. Amniocentesis

The second invasive method of sampling, which is considered the gold standard, is amniocentesis [39]. It is a widely used invasive prenatal diagnostic procedure in which a sample of amniotic fluid is obtained transabdominally under ultrasound guidance, typically between 15 and 20 weeks of gestation, by inserting a fine needle through the maternal abdomen into the uterine cavity [40]. The collected fluid contains fetal cells and biochemical substances, allowing for a broad range of analyses, including chromosomal studies, biochemical and enzymatic assays, molecular genetic testing for single-gene disorders, and assessment for fetal infections or neural tube defects [40,41]. The benefits of amniocentesis include its high diagnostic accuracy, which exceeds 99% in experienced centers; the ability to detect a wide spectrum of genetic and metabolic disorders; and its established safety profile when performed by skilled practitioners [42,43]. Risks associated with the procedure are generally low but include miscarriage, with recent studies reporting attributable risks between 0.1% and 0.5% in experienced hands, amniotic fluid leakage occurring in 1–2% of cases, transient vaginal spotting or abdominal pain, and very rarely, infection or fetal injury [40,43,44]. Performing amniocentesis before 15 weeks is associated with an increased risk of complications such as fetal limb abnormalities, including clubfoot, and is therefore not recommended [44]. In summary, amniocentesis remains a cornerstone of prenatal diagnosis, particularly for chromosomal and single-gene disorders like SMA, offering a favorable balance of diagnostic benefit and procedural safety when appropriately indicated and performed [42,43,44].

#### 2.1.3. Molecular Diagnostic Methods Following Invasive Sampling

Following these sampling methods, there are several molecular methods through which to perform prenatal genetic testing, which are described in the following section.

Multiplex ligation-dependent probe amplification (MLPA) has become the gold standard for detecting *SMN1* gene deletions. The method allows for quantification of deletion in exons 7 and 8 and copy numbers in the *SMN1* and *SMN2* genes, which is essential for distinguishing carriers from affected individuals [45,46]. The MLPA assay significantly improves SMA diagnosis by enabling simple and rapid detection of the main genetic defect of SMA, as well as evaluation of the *SMN2* copy number, which is crucial for predicting disease severity and determining the extent of deletion in 5q13.2 [6]. Although this method is considered the gold standard for the clinical early detection of SMA, some limitations have been described in a study involving 21 families at high risk of SMA. MLPA identified 14 fetuses as carriers and 1 fetus with a homozygous deletion. However, a limitation of MLPA lies in its inability to detect mutations outside the pre-designed probe-binding regions, which can lead to false-negative results [47].

Another molecular method for prenatal SMA diagnosis is real-time quantitative polymerase chain reaction (qPCR) and droplet digital PCR (ddPCR), which offer enhanced sensitivity in determining *SMN1* gene copy number. Quantitative real-time PCR (qPCR) is considered the gold standard in molecular diagnostics due to its high sensitivity, specificity, and relatively rapid turnaround time, making it suitable for quantifying gene expression and detecting genetic variants [48]. Droplet digital PCR (ddPCR) further enhances sensitivity and precision by enabling the absolute quantification of nucleic acids without reliance on calibration curves, which is particularly advantageous for detecting low-abundance targets and rare genetic variants [49].

The main advantages of these methods include their robustness, reproducibility, and applicability to a wide range of diagnostic scenarios [50]. However, qPCR can be affected by variability due to reference standards and reaction conditions, while ddPCR, although more precise, typically requires more complex instrumentation and higher costs [51]. In a study conducted on 138 samples, ddPCR demonstrated complete consistency with the MLPA method, while providing additional advantages in detecting mosaicism and rare genetic variants through absolute quantification without dependence on calibrators. In another more recent study, ddPCR yielded results consistent with those of MLPA in 95% of samples, making it a more suitable method for variant testing [52,53]. The particular value of these methods is evident in their ability to provide a more precise determination of the *SMN2* gene copy number, which may assist in predicting the clinical phenotype [54].

The most robust invasive method in this range of molecular prenatal diagnostics is for cases without a clear deletion of the *SMN1* gene; a combination of long-range PCR and sequencing is employed to identify point mutations. In a Chinese study involving 419 patients, 3.6% of cases exhibited a homozygous deletion of exon 7 without changes in exon 8, necessitating additional sequencing to confirm the diagnosis. This methodology has proven particularly useful in populations with a higher prevalence of atypical genetic variants [27].

### 2.2. Non-Invasive Methods

In contrast to invasive sampling methods, non-invasive approaches for obtaining fetal genetic material have revolutionized prenatal testing by dramatically enhancing both efficacy and safety outcomes. Among these breakthrough methodologies, liquid biopsy through venipuncture of circulating maternal blood has emerged as a cornerstone technique, enabling the isolation of small nucleosomal fragments known as cell-free fetal DNA (cffDNA) from the plasma fraction.

#### 2.2.1. Liquid Biopsy

Non-invasive prenatal testing (NIPT) has consequently gained widespread clinical adoption, with Yang and colleagues demonstrating in their seminal 2015 study that SRY gene amplification results were equivalent between cffDNA and amniotic DNA testing, though they do require optimized centrifugation conditions and commercially available DNA isolation kits [55]. While cffDNA isolation initially presented formidable downstream testing challenges due to low abundance, genomic DNA dilution, and maternal DNA background interference, innovative isolation methods coupled with chemical sample stabilization have yielded representative and stable specimens suitable for transport without requiring in-house testing protocols [56]. Beyond sampling simplicity, the paramount advantage lies in early specimen collection, which facilitates prompt diagnosis and potentially enables fetal-stage therapeutic interventions [55,56].

#### 2.2.2. Molecular Diagnostic Methods Following Non-Invasive Sampling

Among emerging downstream applications, relative haplotype dose (RHDO) represents a pioneering approach utilizing cffDNA to determine pathogenic haplotype inheritance, as exemplified by the NIPSIGEN study, where six SMA carrier pregnancies achieved 100% accuracy in fetal status determination [57]. Although highly precise, RHDO requires comprehensive family haplotyping (including parents and the proband) and targeted sequencing, necessitating sophisticated bioinformatics for background noise elimination, contamination control, and appropriate DNA library preparation—factors that may limit clinical implementation due to accessibility and cost considerations [58].

A more recent downstream methodology involves targeted sequencing using next-generation sequencing (NGS) technology, which enables the detection of structural variants without requiring haplotype data while enhancing sensitivity, particularly in pregnancies with low fetal DNA concentrations. This approach has been recently adapted for rapid, “plug-and-play” SMA patient screening solutions; however, limitations include complex data analysis requirements and restricted laboratory availability [59].

Building upon the insights provided by the two preceding sections, Table 3 presents a comprehensive overview of genetic material sampling methods, their respective invasiveness levels, and the downstream analytical techniques utilized for genetic variant testing.

### 2.3. Increasing Clinical Potential of Prenatal Testing

Previously, prenatal testing for many diseases was used to help parents make an informed decision regarding the birth of children, especially in the setting of known genetic diseases in the family. An interesting recent paper covering this topic revealed that 91% of respondents (60% patients) supported prenatal testing and that 81% of patients felt their diagnosis was delayed [60], indicating a clear and positive opinion. The main benefits of prenatal testing can be early knowledge and reproductive choice, facilitating early intervention, mitigating risk, and aiding family planning [61].

One of the most significant benefits of prenatal testing is that it empowers families with accurate information. For carrier couples, knowing the SMA status early in pregnancy allows for informed decision making. Some may choose to prepare medically and emotionally for a child with SMA. In contrast, others may consider termination in cases of the most severe SMA (Type I), especially in regions or cultures where this is acceptable. Still, in the United States, many prenatal genetic counsellors do not discuss potential treatment with families, even though there is growing awareness and screenings have been implemented in many countries worldwide [62].

**Figure 1:**
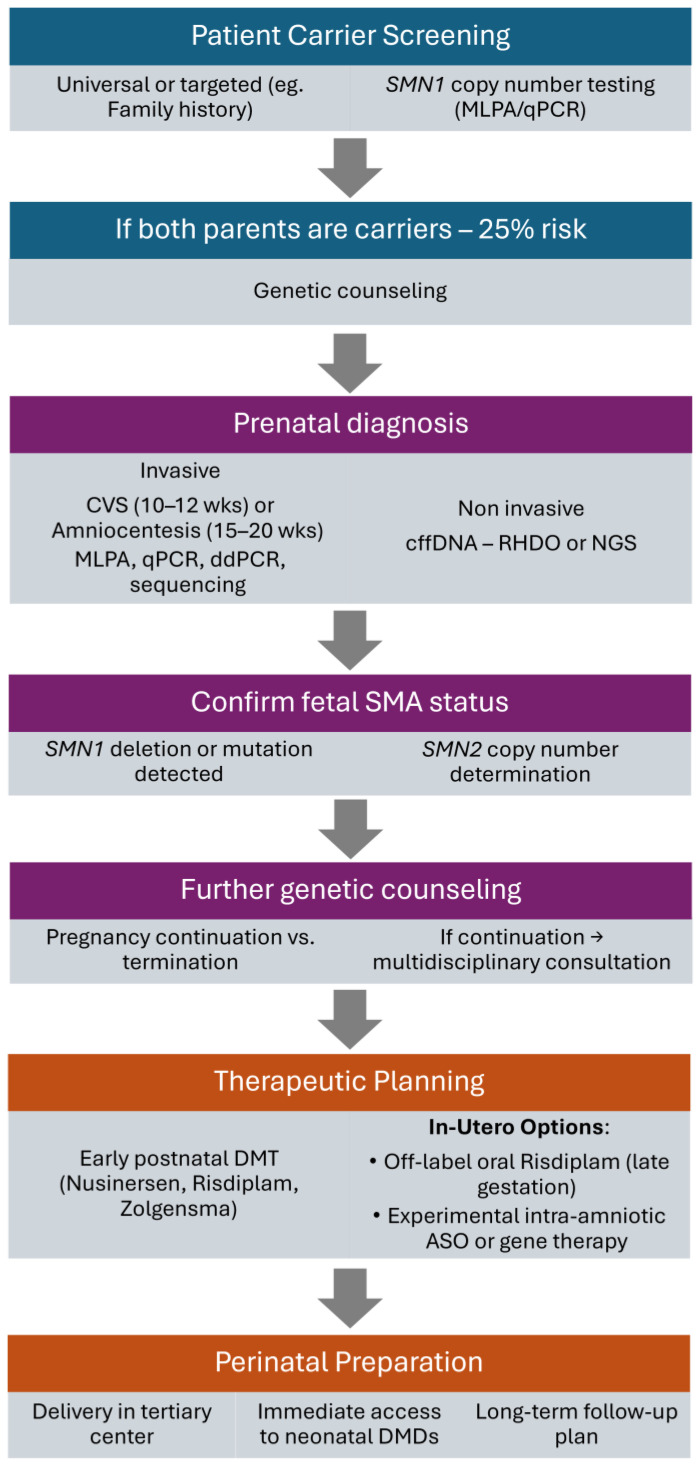
Flowchart of procedures relating to prenatal testing and therapy. Abbreviations: SMA, spinal muscular atrophy; *SMN1/SMN2*, survival motor neuron 1/2 genes; MLPA, multiplex ligation-dependent probe amplification; qPCR, quantitative polymerase chain reaction; ddPCR, droplet digital polymerase chain reaction; CVS, chorionic villus sampling; cffDNA, cell-free fetal DNA; RHDO, relative haplotype dosage; NGS, next-generation sequencing; DMD, disease-modifying drug; ASO, antisense oligonucleotide.

It would be important to implement a stepwise clinical framework for prenatal management of SMA (Figure 1). Beginning with carrier screening and genetic counseling, the process advances through prenatal diagnostic options, both invasive (chorionic villus sampling and amniocentesis) and non-invasive (cell-free fetal DNA analysis). Upon confirmation of fetal SMA and SMN2 copy number, families engage in multidisciplinary decision making regarding pregnancy continuation and therapeutic strategies. If continued, early postnatal treatment or in utero therapy, including off-label or experimental options, may be considered in specialized centers.

With the advent of disease-modifying SMA therapies (such as nusinersen, risdiplam, and onasemnogene gene therapy), early detection of SMA has a direct impact on health outcomes. Multiple studies have found that treating infants before or right after symptom onset leads to dramatically improved motor function and survival [63,64,65]. For example, presymptomatic infants with 3 *SMN2* copies who received nusinersen shortly after birth achieved near-normal motor milestones, and infants treated within the first 6 weeks of life fared better than those treated after 6 weeks [66]. Therefore, a prenatal diagnosis can facilitate immediate access to therapy after delivery. Babies known to have SMA can receive prompt interventions, which can prevent irreversible motor neuron loss. This early treatment window is critical; SMA newborn screening programs aim to start therapy within days to weeks of birth for the same reason. This can substantially improve quality of life and developmental outcomes for the affected child [67].

## 3. Advances in In Utero Therapies: Clinical Innovations, Genetic Interventions, and Health System Perspectives

As mentioned earlier, the groundbreaking case of risdiplam administration for type 1 SMA reported in the early 2025 represents a significant milestone in the evolving field of fetal therapy [24]. In utero therapy itself has progressed substantially over recent decades, as it has evolved from experimental procedures to established treatments for select conditions, offering hope for those previously considered untreatable until after birth.

When it comes to fetal (i.e., in utero) therapy, beginnings can be traced to the early 1980s, when practitioners from various institutions gathered to discuss emerging approaches to prenatally diagnosed congenital anomalies [68,69]. From then onward, this field has expanded rapidly, including the development of Fetal Therapy Centers (FTCs) worldwide, which was made possible by advances in prenatal diagnosis, fetal imaging, instrumentation, and interventional techniques [70].

Modern fetal therapy encompasses a spectrum of interventions that vary in invasiveness and application [71]. Among invasive procedures, ultrasound-guided needle procedures are considered minimally invasive and are commonly used for both diagnostic and interventional purposes [72]. Fetoscopic procedures involve the insertion of a small laparoscope into the uterus to visualize the fetus and placenta. They have been developed for laser treatment of twin–twin transfusion syndrome [73], tracheal balloon occlusion for congenital diaphragmatic hernia [74], and the ultrasound-guided fetal delivery of mesenchymal stem cells [75]. Open fetal surgeries are more invasive procedures that require a hysterotomy [69]. These open fetal surgical techniques have been predominantly used for myelomeningocele [76], for some congenital pulmonary airway malformations [77], and for the treatment of sacrococcygeal teratomas [78]. A specialized delivery technique called ex utero intrapartum treatment (EXIT) is performed by maintaining the maternal placental circulation while establishing an airway in fetuses with airway obstructions [79].

However, the newest frontier in fetal therapy includes some emerging genetic and cellular therapies, including in utero gene therapy, stem cell transplantation, prenatal enzyme replacement therapy (ERT), and small molecule drug administration.

### 3.1. Prenatal Intervention in Spinal Muscular Atrophy: A New Frontier in Fetal Therapy

The February 2025 case of risdiplam administration represents a significant advancement in treating SMA prenatally [24]. This case, conducted at St. Jude Children’s Research Hospital, involved administering risdiplam (5 mg/day) to the mother from 32 weeks and 5 days of gestation until delivery. The drug was detected in both amniotic fluid (33% of maternal plasma level) and cord blood (69%), confirming its passage through the placental barrier. More than two years after birth, the child has shown no clinical signs of SMA progression.

In lieu of this case, a group of researchers at the University of California, San Francisco, and Johns Hopkins University have demonstrated promising results in SMA animals using antisense oligonucleotides (ASOs) delivered via amniotic fluid to treat SMA prenatally [80]. Namely, Borges and colleagues administered prenatal treatment with ASOs, delivered via intra-amniotic injection, which improved outcomes in two severe SMA mouse models. Furthermore, in a fetal lamb model, they confirmed the feasibility of this delivery method, achieving broad ASO distribution, including to the central nervous system (CNS), even though therapeutic brain levels were reached in only some of the experimental animals. These results suggest that intra-amniotic delivery could be a viable prenatal treatment route for SMA, but further refinement is needed before clinical application (Table 4).

### 3.2. In Utero Gene Editing: Correcting Genetic Disorders Before Birth

In utero gene editing aims to correct genetic mutations before birth using CRISPR-based tools, revolutionary genome-editing technologies that allow precise modification of DNA. Preclinical studies in animal models show promise for treating conditions like beta-thalassemia and cystic fibrosis, with the fetal stage offering advantages like immune tolerance and access to developing organs [82]. Human trials are still in early stages. In a landmark case, in May 2025, a baby born with a severe urea-cycle disorder that leads to accumulation of ammonia and death in about half of infants affected was discovered to carry an ultra-rare genomic defect [83]. This case is the first example of a “CRISPR-for-one” therapy, highly customized to an individual patient’s genetic sequence and unlikely to be used for others due to its specificity. The therapy was developed in just six months, demonstrating the potential for rapid, personalized gene-editing treatments for rare diseases that currently lack effective therapies. At the moment of this report, the treated patient is showing signs of clinical benefit.

In utero CRISPR gene editing in humans has not yet been reported in clinical trials. However, in a study by the researchers at the University of California, Davis and Berkeley, a high-efficiency method for in utero gene editing in embryonic mouse brains was demonstrated [84]. By using the acid-degradable PEGylated lipid nanoparticles, they were able to deliver CRISPR-associated mRNA and guide RNAs directly into the fetal brain via intracerebroventricular injection at embryonic day 15.5. This approach achieved widespread gene editing in neural progenitor cells, affecting over 40% of cortical neurons and 60% of hippocampal neurons by 10 weeks after birth. Despite the evident promise, especially in preventing neurodevelopmental disorders such as Angelman syndrome and Rett syndrome, in utero gene editing is still in the experimental stage and has not yet been applied in human prenatal clinical settings.

### 3.3. Comparative Approaches to In Utero Therapies in Genetic Diseases: Lessons from In Utero Enzyme Replacement Therapy for Lysosomal Storage Disorders

While SMA and lysosomal storage disorders (LSDs) represent distinct categories of genetic disease, recent advancements in in utero therapy for LSDs offer a valuable framework for exploring prenatal interventions in SMA. The progress in in utero ERT for LSDs underscores the feasibility and potential benefits of fetal intervention in neurodegenerative diseases. By comparing these approaches, we can better understand the logistical, immunological, and ethical challenges of prenatal therapy and apply these insights to SMA. Thus, in utero ERT serves as a compelling model and catalyst for the development of fetal therapies in SMA, highlighting the urgency of earlier therapeutic windows.

Prenatal ERT is an emerging approach aimed at treating lysosomal storage disorders and other enzyme deficiency conditions before birth [85]. These disorders, like SMA, show rapid prenatal or perinatal disease progression, making in utero intervention theoretically more effective. Preclinical studies, primarily in animal models, have shown that in utero ERT can prevent or significantly reduce disease manifestations by delivering functional enzymes during critical periods of fetal development [86]. Conditions such as Pompe disease, Hurler syndrome, and Gaucher disease have been among the initial targets for prenatal intervention due to their severe early-onset forms.

In terms of delivery techniques, intra-amniotic, intraperitoneal, and intravenous routes have been explored. Furthermore, studies have indicated that repeated prenatal dosing can lead to enzyme uptake in key organs (including the liver, heart, and the CNS) and that early exposure may promote immune tolerance, potentially reducing the risk of anti-enzyme antibody formation that often complicates postnatal therapy. Despite the promise of prenatal ERT, there are several challenges: the need for precise fetal diagnosis, optimizing dosing regimens, ensuring placental or systemic enzyme uptake, and managing procedural risks.

The PEARL trial, the first-in-human clinical study investigating the safety and efficacy of in utero ERT for fetuses with lysosomal storage diseases [63,87,88,89], was designed to evaluate eight different lysosomal storage disorders under a single investigational new drug application, including mucopolysaccharidosis (MPS) types 1, 2, 4a, 6, and 7; infantile-onset Pompe disease; neuronopathic Gaucher disease (types 2 and 3); and Wolman disease.

Thus far, five cases have been reported. The first published case was the Pompe disease case [90], in which the baby was treated prenatally by alglucosidase alfa (20 mg/kg of estimated fetal weight, at 24–34 weeks gestation, six doses). The baby was born to term and had a normal heart, normal motor functions, and early resolution of antibodies, unlike other patients with the disease. The second patient had MPS type 2 (Hunter syndrome), received five doses of ERT, and was born to term with normal biomarkers at birth, representing a significant improvement compared to typical untreated cases [91]. The third patient with MPS type 1 received three in utero doses of ERT and was born to term and also had normal biomarkers at birth. Lastly, according to interim data presented at the 21st World Symposium in 2024 [92], two additional patients (the fourth and fifth cases) have been treated as part of the PEARL trial, though specific details about their conditions and outcomes were not fully disclosed in the available reports.

### 3.4. Pharmacoeconomic and Health Technology Assessment Perspectives

The rapid evolution of DMDs for SMA has brought significant clinical gains but has also raised complex economic and policy challenges. Orphan medicines such as nusinersen, risdiplam, and onasemnogene abeparvovec are among the most expensive therapies globally, prompting intense scrutiny regarding their cost-effectiveness and long-term value. While these therapies offer substantial improvements in survival, motor function, and quality of life, their high upfront costs necessitate critical evaluation within health technology assessment (HTA) frameworks. As emphasized by Belančić et al. in their systematic literature review [22], onasemnogene abeparvovec demonstrates the most favorable cost-effectiveness profile (if administered under a standard single-dose treatment once in a lifetime scenario), followed by risdiplam, with nusinersen being the least cost-effective option. Importantly, the timing of intervention plays a decisive role in value optimization. Initiating therapy in the presymptomatic (or now even prenatal phase) has been shown to be the most cost-effective scenario, aligning clinical and economic priorities by reducing the burden of irreversible neurodegeneration and long-term supportive care.

In this context, in utero treatment represents not only a clinical frontier but also an economic opportunity, provided that safety and efficacy thresholds are met. If one is constructing an economic model for “SMA in utero therapy”, it is essential to include the potential costs of gestational complications, both direct and indirect (e.g., maternal leave, psychosocial stress, and emotional burden). These elements are critical to capturing the full spectrum of health outcomes and real-world costs. Nevertheless, pharmacoeconomics is only one dimension of HTA. For rare diseases and orphan medicines, other domains—including clinical benefit, safety, ethical justification, social value, and impact on caregiver quality of life—must be integrated into decision making [93,94,95,96]. Ethical and societal considerations, in particular, often carry significant weight in the appraisal of rare disease interventions, underscoring the need for a holistic multidimensional evaluation approach. Policymakers and payers should therefore consider the full HTA spectrum when assessing early treatment strategies, especially as prenatal and in utero interventions begin to shift the paradigm of care for genetic diseases like SMA [97].

## 4. Regulatory and Ethical Considerations in Fetal Therapy

Fetal therapy is a rapidly evolving field that presents distinct regulatory and ethical challenges. As science progresses, frameworks and ethical principles must adapt to ensure safe and equitable care for both pregnant individuals and their unborn children. The regulatory landscape governing fetal therapy is intricate and still under development.

Currently, the U.S. Food and Drug Administration (FDA) does not maintain specific regulations mandating the inclusion of pregnant populations in clinical trials; however, general protections for human subjects, including those under 45 CFR 46 Subpart B, do apply. In recent years, the FDA has issued guidance encouraging the inclusion of pregnant populations in clinical research when scientifically and ethically appropriate, recognizing the longstanding underrepresentation of this group and the resulting gaps in evidence-based care during pregnancy [98]. This evolving stance is reflected in the designation of select prenatal therapies as Breakthrough Therapies, acknowledging their potential to address serious maternal or fetal conditions. In the European Union, the EMA regulates in utero (fetal) therapies within the existing Advanced Therapy Medicinal Products (ATMPs) and pediatric frameworks. While no dedicated guidance specifically addresses in utero interventions, these frameworks explicitly accommodate fetal therapies and emphasize rigorous safety, quality, and ethical standards. Across both regulatory landscapes, the emphasis remains on careful risk–benefit assessment and the ethical inclusion of pregnant individuals in research where appropriate [99].

One of the primary ethical dilemmas of fetal therapy involves balancing the health and autonomy of the pregnant individual with potential benefits and risks to the fetus, and most experts agree that ethical decision making in this context should give equal consideration to maternal well-being, fetal outcomes, and maternal autonomy [100].

Equity is another ethical concern, as access to fetal therapy can be limited by geographic location and economic status with the raising disparity in healthcare delivery and access to innovative medical treatments [101]. Finally, the development of gene-editing technologies introduces new ethical dimensions, such as where to draw the boundaries of prenatal intervention and what the long-term implications of altering the human genome before birth might be.

## 5. Future Considerations and Recommendations for Research

Prenatal diagnosis of SMA has undergone revolutionary advancement through the development of quantitative PCR methodologies and non-invasive techniques based on cffDNA analysis. While MLPA continues to serve as the cornerstone of invasive diagnostic approaches, emerging technologies such as ddPCR and RHDO present novel opportunities for personalized clinical management strategies. The primary challenges that persist include reducing the cost burden of non-invasive testing platforms and enhancing clinician education regarding the genetic complexities inherent to SMA pathophysiology. Future endeavors should prioritize the integration of prenatal and newborn screening protocols within global healthcare infrastructures, as this comprehensive approach will undoubtedly present new challenges while simultaneously optimizing prenatal testing efficacy and therapeutic outcomes. The forthcoming landscape promises significant optimization of non-invasive methodologies, particularly through the development of advanced cffDNA analysis platforms capable of reducing the required fetal fraction threshold from 4% to 2% [59]. Concurrently, the refinement of downstream analytical approaches—specifically the integration of NGS and ddPCR technologies with artificial intelligence algorithms—will substantially enhance the accuracy, accessibility, and operational simplicity of these diagnostic modalities [102,103,104]. Such technological convergence would ultimately facilitate the implementation of universal prenatal screening programs, marking a paradigm shift toward comprehensive population-based genetic health screening.

While early therapy after birth is showing promising results, prenatal therapy has groundbreaking potential. A case report published in the New England Journal of Medicine presented positive results of in utero prenatal risdiplam therapy carried out over 30 months after birth [24]. Preclinical mouse models show that antisense oligonucleotide treatment with intra-amniotic administration can significantly improve outcomes [80]. This is true for numerous genetic diseases, such as cystic fibrosis [105]. Furthermore, there is an example of onasemnogene abeparvovec treatment in two prematurely born twins, which has shown excellent treatment outcomes. Interestingly, the patients had one present positive genetic modifier, which likely influenced the outcome and highlights the importance of modifiers in clinical practice [106]. There is still no standard way of administering vector-based therapies in utero, although it is likely that the administration should be via an intraamniotic injection [107]. Finally, there is also the option of combining multiple forms of genetic therapy, which is a proven approach in SMA [108]. It is feasible to envision starting oral-based therapy during gestation, as in the previously reported case report, and then combining it with onasemnogene abeparvovec.

Even though several challenges remain, the routine clinical translation of this form of therapy is nearing clinical practice, with a clear recommendation for clinical trials. These trials will require a firm prenatal diagnosis of the disease, and this could further outweigh the potential risks [109].

## Figures and Tables

**Table 1:** Natural history-based classification of spinal muscular atrophy (SMA): age of onset, motor function, and prognosis before therapeutic intervention.

SMA Type	Age at Symptom Onset	Maximum Motor Function Achieved	Prognosis
0	Prenatal/fetal	Nil	Poor; usually die before 6 months
1	<6 months	Non-sitter	Poor; usually die before 2 years
2	7–18 months	Sit independently	Expected to live into their twenties and beyond
3	>18 months	Walker	Progressive weakness, motor disability, normal lifespan
4	10–30 years	Walker	Weakness of the lower extremities, normal lifespan

**Table 2:** Summary of approved treatments for spinal muscular atrophy (SMA).

Treatment	Year of EMA Approval	Type of Molecule	Administration Route	Pros	Cons
Nusirsen	May 2017	Antisense oligonucleotide	Intrathecal	First approved SMA therapy; proven efficacy in clinical trials (ENDEAR, CHERISH)	Requires repeated lumbar punctures; invasive administration
Onasemnogene abeparvovec	May 2020 (conditional); May 2022 (full)	Gene replacement therapy	Intravenous	One-time treatment; targets genetic root cause; early intervention shows strong benefit (START, STR1VE)	High cost; potential liver-related side effects
Risdiplam	March 2021	Oral splicing modifier	Oral	Convenient oral administration; effective across broad age range (FIREFISH, SUNFISH)	Daily dosing required; newest (long-term data still emerging)

**Table 3:** Sampling method, invasiveness, and downstream methods of actual prenatal testing.

Sampling Method	Invasiveness	Downstream Method
Chorionic villus sampling (CSV)	Invasive	MLPA, qPCR, ddPCR
Amniocentesis		
Liquid biopsy (cffDNA)	Non-invasive	RHDO, Deep sequencing, NGS

Abbreviations: MLPA, multiplex ligation-dependent probe amplification; qPCR, quantitative polymerase chain reaction; ddPCR, digital droplet polymerase chain reaction; cffDNA, cell-free fetal DNA; RHDO, relative haplotype dosage; NGS, next-generation sequencing.

**Table 4:** Summary of human and animal studies on in utero therapy for spinal muscular atrophy.

Study/Institution	Model	Therapy	Delivery Route	Timing	Key Findings
St. Jude Children’s Research Hospital [24]	Human fetus	Risdiplam (5 mg/day)	Oral to mother transplacental	Starting at 32 weeks + 5 days gestation until delivery + newborn continued treatment from 8 days of age	Risdiplam detected in amniotic fluid and cord blood; no clinical SMA signs in child at >2 years; some congenital abnormalities were noted that likely occurred before treatment initiation
Borges et al. (UCSF & Johns Hopkins) [80]	Mouse (2 models)	ASO	Intra-amniotic injection	Mid-gestation	Significant improvement in SMA phenotype and survival
Borges et al. (UCSF & Johns Hopkins) [80]	Fetal lamb (feasibility and safety study)	ASO	Intra-amniotic injection	Mid-to-late gestation	Broad CNS distribution observed; therapeutic brain levels achieved in some cases; delivery route feasible but needs optimization
UC Davis & UC Berkeley	Mouse embryo	CRISPR- Cas9 gene editing	Intracerebroventricular injection	E15.5	>40% gene editing in cortical neurons and >60% in hippocampus; proof of concept for in utero CNS gene editing
Rashnonejad et al. [81]		AAV vectors (AAV9-SMN)	Intracerebroventricular injection	E14.5–E15	Increased median survival rates; histological improvements; study confirmed that prenatal was more effective than postnatal intervention

Abbreviations: AAV, adeno-associated virus; ASO, antisense oligonucleotides; E, embryonic day.

## Abbreviations

The following abbreviations are used in this manuscript

ASOs	Antisense Oligonucleotides
cffDNA	Cell-free Fetal DNA
CNS	Central Nervous System
CRISPR	Clustered Regularly Interspaced Short Palindromic Repeats
CVS	Chorionic Villus Sampling
ddPCR	Digital Droplet PCR
DMDs	Disease-Modifying Drugs
EMA	European Medicines Agency
ERT	Enzyme Replacement Therapy
EXIT	Ex Utero Intrapartum Treatment
FDA	Food and Drug Administration
FTCs	Fetal Therapy Centers
MLPA	Multiplex Ligation-Dependent Probe Amplification
MPS	Mucopolysaccharidosis
NGS	Next-Generation Sequencing
NIPT	Non-Invasive Prenatal Testing
PCR	Polymerase Chain Reaction
qPCR	Quantitative Polymerase Chain Reaction
RHDO	Relative Haplotype Dosage
SMA	Spinal Muscular Atrophy
SMN	Survival Motor Neuron
*SMN1*	Survival Motor Neuron 1 (gene)
*SMN2*	Survival Motor Neuron 2 (gene)
